# Investigation of bactericidal effect of a mid-infrared free electron laser on *Escherichia coli*

**DOI:** 10.1038/s41598-022-22949-9

**Published:** 2022-10-27

**Authors:** Toshizo Toyama, Jun Fujioka, Kiyoko Watanabe, Ayaka Yoshida, Takaaki Sakuma, Keitaro Inaba, Takayuki Imai, Takashi Nakajima, Koichi Tsukiyama, Nobushiro Hamada, Fumihiko Yoshino

**Affiliations:** 1 Department of Oral Microbiology, Kanagawa Dental University, 82 Inaoka-cho, Yokosuka, Kanagawa 238-8580 Japan; 2 IR FEL Research Center, RIST, Tokyo University of Science, 2641 Yamazaki, Noda, Chiba 278-8510 Japan; 3 Department of Liberal Arts Education, Kanagawa Dental University, 82 Inaoka-cho, Yokosuka, Kanagawa 238-8580 Japan; 4 Department of Dental Education Liberal Arts/Dental Education Institute, Kanagawa Dental University, 82 Inaoka-cho, Yokosuka, Kanagawa 238-8580 Japan; 5 Sakuma Dental Clinic, 15-1 Yashikinaka-Aza, Moriai, Fukushima, Fukushima 906-8003 Japan; 6 Department of Applied Physics, Faculty of Science, Tokyo University of Science, 6-3-1 Niijuku, Katsushika-ku, Tokyo, 125-8585 Japan; 7 Department of Pharmacology, Kanagawa Dental University, 82 Inaoka-cho, Yokosuka, Kanagawa 238-8580 Japan

**Keywords:** Microbiology, Antimicrobials

## Abstract

The rapid increase in the number of bacteria that are resistant to many commonly used antimicrobial agents and their global spread have become a major problem worldwide. In particular, for periodontal disease, which is a localized infection, there is a growing need for treatment methods that do not primarily involve antimicrobial agents, and antimicrobial photodynamic therapy (aPDT) is attracting attention. In this study, the bactericidal effects of a mid-infrared free electron laser (MIR-FEL) on *E. coli* were investigated as a basic study to examine the applicability of MIR-FELs, which can selectively excite molecular vibrations due to their wavelength tunability, to aPDT. The optimal irradiation wavelengths to be examined in this study were determined from the infrared spectrum of the bacteria, which was obtained using Fourier transform infrared spectroscopy. Five irradiation wavelengths (6.62, 6.88, 7.14, 8.09 and 9.26 µm) were selected from the FT-IR spectrum, and we found that the bactericidal effects at a wavelength of 6.62 µm were markedly stronger than those observed at the other wavelengths. At this wavelength corresponding to the Amide II band, the bacterial survival rate decreased significantly as the irradiation time increased. On the contrary, irradiation of a neodymium-doped yttrium aluminum garnet (Nd: YAG) laser at 1.06 µm exhibited no distinct bactericidal effect. No morphological changes were observed after MIR-FEL irradiation, suggesting that a bacterial organelle molecule may be the target of MIR-FEL irradiation, but the exact target was not identified. Furthermore, the temperature change induced in the culture medium by the laser irradiation was ± 1.5 °C at room temperature. These results suggest that the bactericidal effects of MIR-FEL are derived from photochemical reactions involving infrared photons, since *E. coli* is usually killed by heating it to 75 °C for 1 min or longer.

## Introduction

The infrared (IR) free-electron laser (FEL) installed at Tokyo University of Science (TUS) Noda Campus (FEL-TUS) is a high-power pulsed laser. The main FEL-TUS device is a mid-infrared FEL (MIR-FEL) with an oscillation wavelength range in the 5–12 µm, which covers almost the entire molecular fingerprint region^1^. This wavelength range corresponds to the fundamental vibrational frequencies of molecules; therefore, the MIR-FEL can be used to study the photochemical properties of a number of substances, including molecules, organic materials, bio molecules, biological cells, etc. through the selective vibrational excitation^2^. The wide instantaneous bandwidth of MIR picosecond oscillators are particularly appealing since they permit powerful Fourier transform (FT) techniques to be employed, which transfer the burden of precise wavelength calibration from the source to the detection system, while also providing excellent signal-to-noise characteristics and wavelength-independent spectral resolution^3,4^.

The FEL-TUS introduces radiation, which is produced by accelerating electrons at close to the speed of light in a linear accelerator, into a periodic magnetic field and then amplifies the radiation through the interaction between the radiation and an electron beam in a resonator, generating a laser beam^5^. The resultant laser light is characterized by (I) a special pulse structure consisting of macro- and micro-pulses, (II) high brightness, (III) a variable wavelength, and (IV) perfect linear polarization. In addition, the wide wavelength tunability of the FEL-TUS allows selective molecular vibrational excitation, providing an appropriate light source for the dissociation of molecules through vibrational ladder climbing^6^.

In dental practice, neodymium-doped yttrium aluminum garnet lasers (Nd: YAG lasers), which have a typical emission light wavelength of 1064 nm, and erbium-doped (Er): YAG lasers (2940 nm) are frequently employed for sterilization during root canal procedures and the treatment of periodontal disease^7–9^. Chemical disinfection with sodium hypochlorite solution has traditionally been used for root canal treatment^10^, and the application of antibiotic-containing ointment or mechanical removal with a scaler are common treatments for periodontal disease. Recently, the use of lasers for sterilization has attracted attention; however, the lasers used for such procedures have a fixed wavelength, and few appropriate light sources with variable wavelengths are available. In recent years, the sterilization of titanium dioxide implant surfaces by UV lasers and near-infrared sterilization of COVID-19 have been reported^11,12^. However, there have been only a few reports on the sterilization effects of MIR-FELs since 1998^13,14^, although MIR-FELs were expected to be introduced as new medical devices in 2006^15^.

Sterilization is critical for periodontal disease treatment, and various sterilization methods have been reported^16^. Antimicrobial agents are the most common type of drug therapy. However, the rapid increase in the prevalence of bacteria that are resistant to many commonly used antimicrobial agents and their global spread are becoming major problems worldwide^17^. In addition, the pace of development of antimicrobial agents is clearly decreasing, especially for the treatment of periodontal disease, which involves local infections, and there is a growing need for research into treatments with different mechanisms of action^18–20^.

In recent years, photodynamic therapy (PDT) has been developed as an alternative treatment for several types of cancer^21^. One of the main advantages of this type of treatment is that it does not have severe side effects, and hence, can be repeated frequently^22^. It has also been used to photo-inactivate Gram-negative/-positive microorganisms for sterilization purposes, which is called antimicrobial photodynamic therapy (aPDT)^23,24^. Against this background, aPDT has been reported to be useful for sterilizing many microorganisms, including both oral pathogens and multidrug-resistant bacteria^25–28^. However, in general, aPDTs require the use of exogenous or endogenous dyes, and few studies have reported that target the intramolecular bonds of bacteria-specific organelles without requiring these dyes.

Many of the building blocks of life are specifically sensitive to radiation from the mid-infrared region, and MIR-FELs are able to selectively excite molecular vibrations^15,29,30^. In addition, if MIR-FEL irradiation has bactericidal effects without the need for a dye, which would differ from the conventional aPDT technique, it may be possible to use them to develop a simpler disinfection method. Therefore, in this study we performed a basic study of the bactericidal effects of MIR-FEL irradiation on *Escherichia coli*, with the goal of exploring the possibility of using MIR-FELs as new aPDT devices for infectious disease control.

## Methods

### Bacterial strain and culture conditions

The *E. coli* HB-101 strain was used as an indigenous Gram-negative bacterium in this study. The *E. coli* were aerobically cultured in brain heart infusion broth (BHI broth; Beckton Dickinson Co., Sparks, MD, USA) with 1.5% Bacto agar (Beckton Dickinson) for 24 h.

### Laser light irradiation

#### (I) MIR-FEL irradiation and IR absorption spectroscopy of *E. coli*

The MIR-FEL, which was operated at 5 Hz, was reflected vertically using a gold-coated mirror and focused with a BaF_2_ lens (Pier-optics Co., Ltd., Gunma, Japan), and the optical path was adjusted so that the entire bacterial solution was subjected to irradiation. The power of the laser just in front of the sample was ~ 10 mJ/pulse. To determine the optimal MIR-FEL wavelengths to study, the IR spectrum of *E. coli*, which were smeared onto a glass slide and air dried for 15 min, was measured by a conventional FT-IR spectrometer (JASCO FT/IR-6100, JASCO, Tokyo, Japan). The FT-IR measurements were performed using the Attenuated Total Reflection technique (ATR)^31^ with the following measurement parameters: number of scans: 64, resolution: 4 cm^−1^, power: 5–8 mJ/pulse, and measurement range: 4000–800 cm^−1^ (2.5–12.5 µm).

#### (II) Nd: YAG laser irradiation

The light produced using an LS-2137 2-DL (LOTIS II Co., Minsk, Belarus) Nd: YAG laser operated at 5 Hz was filtered out at 1064 nm by passing it through a bandpass filter and then reflected vertically using a mirror. The optical path was adjusted so that the entire bacterial solution was covered by the irradiation field. The power of the laser just in front of the sample was 10 mJ/pulse.

#### (III) Bactericidal activity

An overnight culture of the bacteria (100 µL) with an absorbance value of 1.0 at a wavelength of 600 nm was centrifuged and suspended in 10 µL of physiological saline (Otsuka Pharmaceutical Co., Ltd., Tokyo, Japan). The bacterial suspension was irradiated with one of the lasers for 5, 15, or 30 min. The control samples were not irradiated, but were left for the same length of time as the irradiated samples. After being irradiated, each sample was serially diluted, and 0.1 mL of the diluted sample was smeared onto BHI agar plates, which were then aerobically cultivated for 24 h. Subsequently, the number of colonies was counted, and the number of viable bacteria was calculated in colony-forming units/mL (CFUs). The survival of bacterial cells was estimated based on the number of viable bacteria by counting the number of CFUs after the cells had been irradiated. To examine the antibacterial effects of laser irradiation, the survival rate was determined as the ratio of the viable cell count in the irradiated group to that in the control group.

### Scanning electron microscope examinations

*E. coli* samples that had been irradiated at each planned wavelength with an MIR-FEL or Nd: YAG laser were fixed with 1% glutaraldehyde in 0.1 M sodium cacodylate buffer for 60 min. After fixation, the samples were washed twice with 0.1 M sodium cacodylate buffer and dehydrated through a graded series of aqueous ethanol solutions (50, 70, 80, 90, and 100%; immersion time per series: 15 min), before being air dried. Then, the samples were coated with a thin layer of platinum using ion sputtering system (JFC-1300, AUTO FINE COATER, Japan Electron Optics Laboratory, Ltd., Tokyo, Japan). The morphological changes of bacterial cells were observed with scanning electron microscope (SEM: JCM-6000Plus, JEOL, Tokyo, Japan).

### Measurement of temperature

To examine the influence of laser irradiation (60 min continuous irradiation) on the temperature of the bacteria, the samples were continuously monitored using an SC620 thermal imaging camera (FLIR Systems Japan K.K., Tokyo, Japan) for 60 min during the irradiation procedure.

### Statistical analysis

Statistical analyses were performed by one-way analysis of variance (ANOVA) followed by Tukey’s test using the BellCurve for Excel software (Ver. 3.21, Social Survey Research Information Co., Ltd., Tokyo, Japan). Differences between groups were considered to be significant at *P* < 0.05.

## Results

### Measurement of the infrared absorption spectrum of *E. coli*

**Figure 1:**
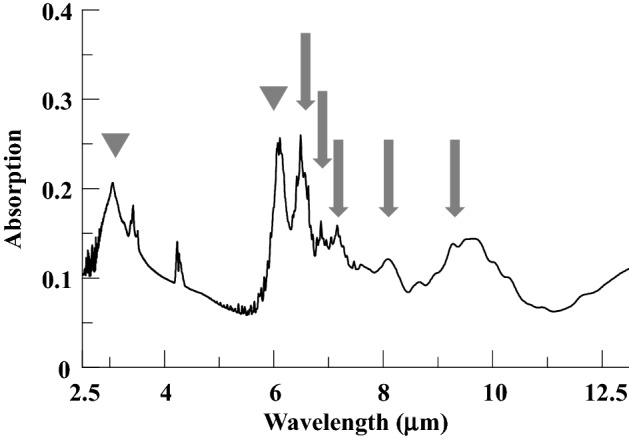
Measurement of the infrared absorption spectrum of *E coli* by infrared absorption spectroscopy. Several distinct absorption peaks are visible. A rather broad peak is seen at ~ 3.0 µm, narrow sharp and strong peaks are observed at 6.00 and 6.62 µm, weak peaks are present at 6.88 and 7.14 µm, and broad peaks can be seen at 8.09 and 9.26 µm. The peaks at around ~ 3.0 and 6.00 µm (marked by grey triangles) were mainly associated with H_2_O. The five wavelengths (marked by grey arrows) were selected as irradiation wavelengths in the current experiment.

The FT-IR spectrum of *E. coli* in the 2.5–12.5 µm wavelength range is shown in Fig. 1. Several distinct absorption peaks were recognized in the abovementioned spectrum. A rather broad peak was seen at ~ 3.0 µm, narrow sharp and strong peaks were observed at 6.00 and 6.62 µm, weak peaks were present at 6.88 and 7.14 µm, and broad peaks were seen at 8.09 and 9.26 µm. According to the IR spectra provided by the NIST Chemistry Reference Database (NIST Chemistry WebBook)^32^, the mid-infrared absorption of H_2_O in the condensed phase exhibits a single sharp peak at around 1648 cm^−1^ (6.07 µm) and a broad peak around 3360 cm^−1^ (2.98 µm). Accordingly, the peaks seen at ~ 3.0 and 6.00 µm (marked by grey triangles in Fig. 1) could be safely assigned to H_2_O, although it is possible that the 6.00-µm band relates to the amide I band of *E. coli* as mentioned later. The other five wavelengths (marked by grey arrows) were selected as irradiation wavelengths for the current study. The corresponding vibrational assignments are listed in Table 1.

**Table 1:** Absorption peaks in the FT-IR spectrum and their vibrational assignments.

Wavelength	Vibrational mode
6.00 µm (1670 cm^−1^)	H_2_O Amide I band
6.62 µm (1510 cm^−1^)	Amide II band
6.88 µm (1453 cm^−1^) 7.14 µm (1426 cm^−1^)	Antisymmetric C–H bending Symmetric COO–carbonate
8.09 µm (1236 cm^−1^)	Amide III band Antisymmetric PO_2_^−^
9.26 µm (1080 cm^−1^)	Symmetric PO_2_^−^

### Bactericidal effects of MIR-FEL irradiation

**Figure 2:**
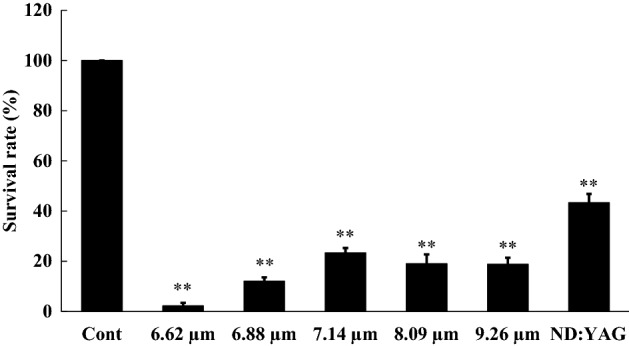
Bactericidal effects of FEL irradiation at various wavelengths on *E. coli.* Comparisons among groups were performed using one-way ANOVA followed by Tukey’s test. Asterisks (**P < 0.01) indicate significant differences from the non-irradiated group or the 15-min MIR-FEL irradiation group.

**Figure 3:**
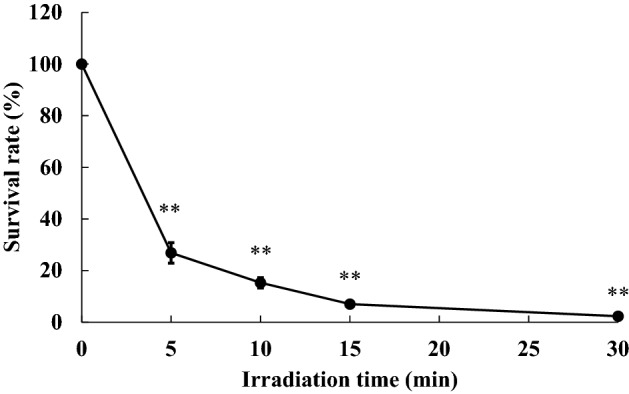
Time-dependent bactericidal effects (viability) of MIR-FEL irradiation at a wavelength of 6.62 µm. Comparisons among groups were performed using one-way ANOVA followed by Tukey’s test. Significant differences are indicated by asterisks (**P < 0.01).

*E. coli* were cultured after MIR-FEL or ND: YAG laser irradiation. Then, the number of CFUs in each group was determined, and the relative survival rate compared with the control was calculated (Table 2 and Fig. 2). Irradiation with the MIR-FEL at 6.62, 6.88, 7.14, 8.09, or 9.26 µm for 15 min significantly reduced the number of viable bacterial cells (*P* < 0.01), resulting in relative viability values of 2.3 ± 1.6%, 12.0 ± 1.1%, 23.2 ± 2.1%, 18.7 ± 1.7%, and 18.6 ± 0.5%, respectively. The bactericidal ability of the MIR-FEL was markedly greater than that of the Nd: YAG laser at all wavelengths (relative viability: 43.3 ± 5.3%, *P* < 0.01), indicating the bactericidal effectiveness of MIR light. In particular, the bactericidal effects seen at a wavelength of 6.62 µm were markedly stronger than those observed at other wavelengths (*P* < 0.01). MIR-FEL irradiation at 6.62 µm significantly reduced bacterial survival in a time-dependent manner (Fig. 3).

**Table 2:** Changes in the number of CFUs induced by MIR-FEL or Nd: YAG irradiation of *E. coli.*

	Control	MIR-FEL (wavelength: µm)					Nd: YAG
		6.62	6.88	7.14	8.09	9.26	
CFUs/mL	5.81 ± 0.38	0.14 ± 0.02*	0.71 ± 0.01*^, †^	1.35 ± 0.01*^, †^	1.09 ± 0.02*^, †^	1.08 ± 0.01*^, †^	2.52 ± 0.05*^, †^

Data are shown as the mean ± SD of 1 × 10^7^ CFUs/mL. Asterisks indicate significant differences (*P* < 0.01) from the control (non-irradiated group), and daggers indicate significant differences (*P* < 0.01) from the 6.62 µm MIR-FEL-irradiated group.

### Scanning electron microscopic images of *E. coli*

**Figure 4:**
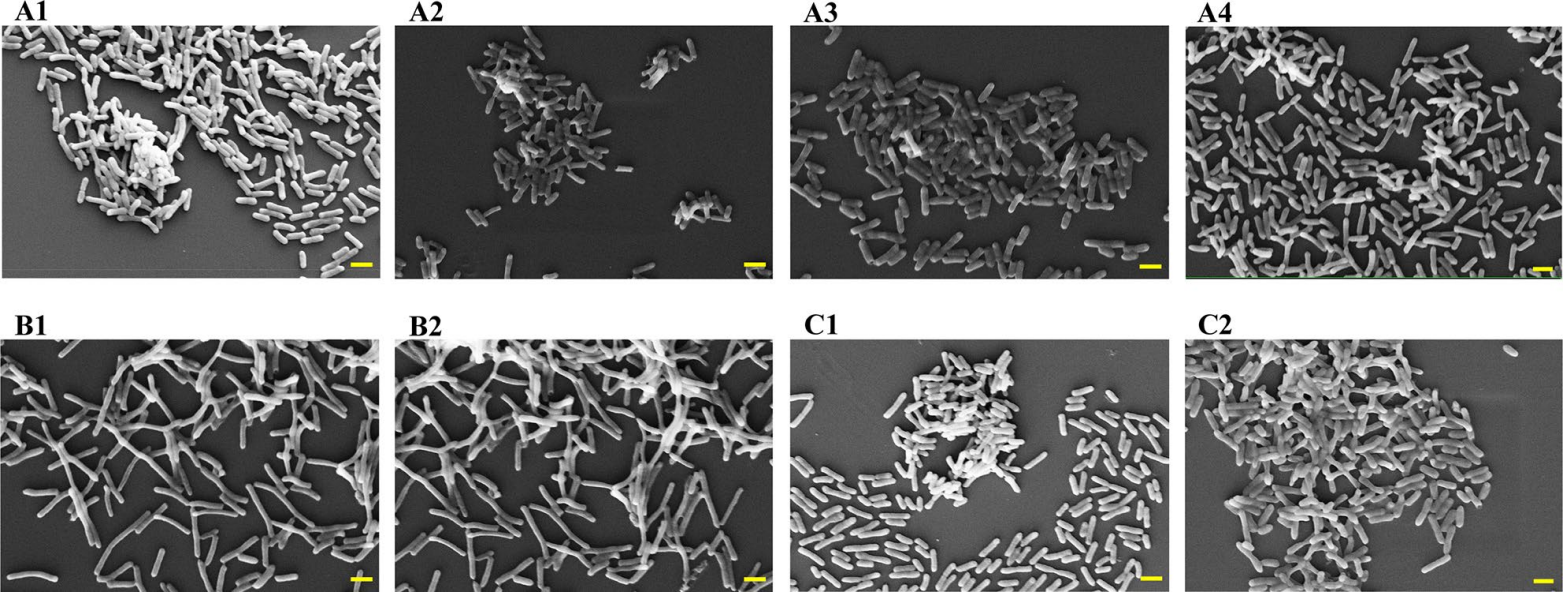
Morphology of *E. coli* cells after MIR-FEL irradiation or ND: YAG laser irradiation. Images of *E. coli* obtained with a scanning electron microscope operating at 10 kV are shown. (**A1**) Untreated group (control); (**A2**) 6.62-µm MIR-FEL irradiation group; (**A3**) 6.88-µm MIR-FEL irradiation group; (**A4**) 7.14-µm FEL irradiation group; (**B1**) control for the 9.26-µm MIR-FEL irradiation group; (**B2**) 9.26-µm MIR-FEL irradiation group; (**C1**) control for the ND: YAG irradiation group; (**C2**) 15-min Nd: YAG laser irradiation group; scale bars, 2 µm.

After 15 min of irradiation at the relevant wavelength, the bacterial cells were examined under a scanning electron microscope. Compared with the non-irradiated group, the number of viable cells was decreased and cell destruction was observed after MIR-FEL irradiation at a wavelength of 6.62 µm. However, no morphological changes were observed after MIR-FEL irradiation at a wavelength of 6.88 µm, and, compared with the non-irradiated group, the number of cells was only slightly decreased after irradiation at an MIR-FEL wavelength of 7.14 µm. The electron microscopic images of the bacterial cells subjected to MIR-FEL irradiation at 9.26 µm or irradiation with the Nd: YAG laser were comparable to those obtained in the non-irradiated group (Fig. 4).

### Measurement of temperature

**Figure 5:**
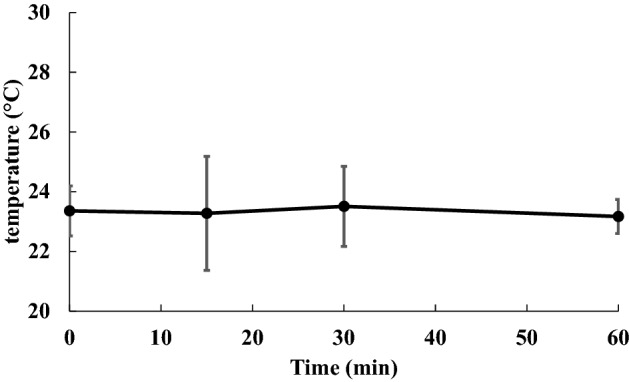
Temperature changes induced in bacterial samples by MIR-FEL irradiation.

The measurement of the temperature of the bacterial samples during the MIR-FEL irradiation was carried out using an SC620 thermographic camera. The mean temperature of the samples remained at room temperature ± 0.12 °C (Fig. 5).

## Discussion

Various sterilization methods are utilized in clinical practice. Of these, lasers are occasionally used for killing oral pathogens in dental practice. Semiconductor lasers, especially Nd: YAG, Er: YAG, and CO_2_ lasers, are the main lasers employed in dental practice^7–9^. In these lasers, a solid or gas is used as a medium, together with a light that oscillates at a single wavelength. The oscillation regions of these lasers are located at ≤ 3 µm or ≥ 10 µm; however, lasers that emit light in the MIR region have not yet been put into practical use. To the best of our knowledge, no reports have been published concerning sterilization of bacteria by resonant vibrational excitation, probably because of the lack of intense wavelength tunable light sources in the finger-print spectral region. Highly efficient irradiation, which can be achieved by laser light irradiation at a wavelength that resonates with a molecular bond, should suppress damage in the surrounding environment. Our group conducts research in the dental field, and this study was a pilot study that attempted to examine whether MIR-FELs could be used to treat periodontal disease. In the present study, the effects of IR radiation in the range of 6–10 µm on *E. coli* were investigated using an MIR-FEL installed at TUS Noda Campus.

Table 1 lists the vibrational assignment of the peaks seen in the FT-IR spectrum of *E. coli*^33–36^. The sharp peak around 6.00 µm corresponds to the amide I (C=O) band^34–36^. As mentioned previously, however, liquid water also exhibits an intense peak around 6.00 µm. Since the water peak around 2.5 µm appears strongly which means the existence of water in the sample, we presume that the peak around 6.00 µm consists of both H_2_O and the amide I band. The sharp peak around 6.62 µm corresponds to the amide II (νC–N coupled with N–H bending of proteins) band^34–36^. Above 7 mm, absorption bands overlap and each peak is not separated, so it seems difficult to assign them to a single vibrational mode. For example, Caine et al.^35^ ascribed the bands around 7 mm to the antisymmetric C–H bending mode (~ 1468 cm^−1^) and symmetric COO– (~ 1400 cm^−1^) of fatty acids and polysaccharides. On the other hand, Acebo et al.^33^ pointed out the possibility of carbonate (1424–1414 cm^−1^). The broad peak around 8.1 mm is assignable to the antisymmetric PO_2_^-^ vibrational mode accompanied by Amide III band^35^. The broad peak around 9.3 mm is a symbol of the symmetric PO_2_^-^ vibrational mode^35^. Among these wavelengths, MIR-FEL irradiation at 6.62 µm (Amide II) produced the greatest post-irradiation inhibition of bacterial growth (Fig. 2). Antimicrobial agents that exert bacteriostatic action include macrolides and tetracyclines, the mechanism of action of which is inhibition of protein synthesis^37,38^. Thus, proteins within microorganisms are important components for their growth and survival. Therefore, it is possible that MIR-FEL irradiation, which showed similar effects, affected all the proteins within the bacteria, resulting in bacteriostatic effects by inhibiting the processes involved in bacterial growth. However, the bacterial target of MIR-FEL was not identified in this study, and the exact target of MIR-FEL irradiation needs to be clarified by quantitative analysis using FT-IR in the future.

Sterilization based on the O–H vibration excitation of water molecules is considered to be similar to the sterilization mechanism of Er: YAG lasers (λ = 2.95 μm), in which the Er: YAG laser evaporates (ablates) the water molecules around the bacterium, and its power causes physical damage to the surrounding molecules. Rauf et al. reported that an energy level of 30 mJ/pulse is required for sterilization by an Er: YAG laser^39^. In conventional aPDT, the use of green LEDs was originally considered to be most desirable in order to maximize the excitation of Rose Bengal and increase the amount of singlet oxygen produced^40,41^. In contrast to the irradiation of near-infrared or visible light, however, the mechanism for sterilization by mid-infrared radiation is definitely different. At a wavelength of 6.62 µm the MIR-FEL hits the Amide II (N–H) band state-selectively, which does not involve the formation of reactive oxygen species, such as singlet oxygen, and theoretically would not cause oxidative damage. On the other hand, as shown in Figs. 2 and 4, the MIR-FEL had bactericidal effects on *E. coli*, but did not cause any morphological changes in the bacteria. These findings suggest that MIR-FEL irradiation may reduce bacterial activity by inhibiting amino acid synthesis through its effects on intermolecular bonds within bacterial organelles.

Although various types of light irradiation, including laser irradiation, may cause thermotoxicity, in the present study the temperature change induced in the culture medium during the irradiation procedure was within ± 0.12 °C (Fig. 5), and the bactericidal effects of these temperature changes can be ignored. This finding strongly suggests that the bactericidal effects of MIR-FELs are derived from IR photons rather than from increases in temperature. The induction of heat-shock protein expression after a temperature shift (from 30 to 42 °C) has been reported to be necessary for improving heat-kill resistance^42^. Although heat-shock analysis of *E. coli* was not performed after irradiation in this study, only a small temperature shift was observed after the MIR-FEL irradiation, and it is unlikely that laser irradiation increases the thermal kill resistance of the target microorganisms; therefore, the adverse effects of performing aPDT multiple times may be insignificant.

In recent periodontal research, pharmacological agents that act on bacterial and host immune responses have been selected as adjuvant treatments. However, none of these antimicrobial agents have become established as gold-standard treatments for periodontal disease^43^. Previous studies have reported that subgingival periodontal bacteria exhibited in vitro resistance to therapeutic concentrations of antibiotics, such as amoxicillin, clindamycin, and doxycycline, which are commonly used for the treatment of periodontal disease^44^. Therefore, since it may not be possible to effectively treat periodontal disease by eradicating Gram-negative bacteria alone, further studies on aPDT are required. However, before MIR-FEL can be applied to the treatment of periodontal disease, it will be necessary to investigate the effects of MIR-FEL on biofilm and *Porphyromonas gingivalis*, which are considered to be the most important factors in the treatment of periodontal disease. The near-infrared Nd: YAG laser (center frequency: 1.064 µm) has a penetration depth of 1–5 mm. The penetration depth of the Er: YAG laser (center frequency: 2.94 µm), which has a lower penetration depth than both the Nd: YAG laser and MIR-FEL, is 1 µm^15^. A typical target for aPDT in dentistry is *P. gingivalis*, and the size of this microorganism is around 1 µm^45^. Therefore, the eradication of *P. gingivalis* by MIR-FEL irradiation is highly feasible and may have clinical applications.

On the other hand, in spite of the fact that aPDT using MIR-FEL shows bactericidal activity against *E. coli* at specific wavelengths, there are still major practical problems, such as its effects on normal cells (safety) when they are irradiated in vivo and the design of the associated irradiation devices, to solve before it can be used in the clinical setting. aPDT could play a role in the treatment of periodontal disease as an adjuvant therapy, and its combination with other periodontal therapies may contribute to significant clinical success^27,28^. For instance, aPDT using blue light has been used to excite endogenous intracellular porphyrins, such as those found in *Propionibacterium acnes*, *Helicobacter pylori*, and *Staphylococcus aureus*^46–48^. Hence, aPDT by MIR-FEL, which does not require the sterilization target to be stained with dyes, may be applicable as a simple aPDT because, unlike conventional aPDT, it can be expected to produce antibacterial effects based on light irradiation alone.

In conclusion, the results of the present MIR-FEL experiments suggest that such lasers may become the cornerstone of a new aPDT that induces intermolecular cleavage through irradiation at a specific wavelength. In addition, aPDT can be performed for repeated infections, and there is little possibility of inducing bacterial resistance, which is not the case when antimicrobial drugs are used repeatedly. These possibilities suggest that MIR-FEL may be used in a new type of aPDT in the future. At present, there are not many reports on the bactericidal effects of MIR-FEL irradiation, although it is very interesting that MIR-FEL irradiation at specific wavelengths exerts bactericidal effects against *E. coli*. It is necessary to investigate the bactericidal effects of MIR-FEL irradiation and the underlying mechanism. However, the size of such devices is the most serious problem limiting the clinical application of MIR-FELs. MIR-FELs consist of a radio frequency electron gun to generate the electron beam, an accelerator tube to accelerate the electron beam, an undulator to generate light by meandering the electron beam, and an optical resonator to amplify and oscillate the light. In particular, the acceleration tube alone is 3 m long^5^, making it difficult to place in a clinic for the sole purpose of sterilization. Further improvement of the system would aid its clinical application.

## Data Availability

The datasets generated during and/or analyzed during the current study are available from the corresponding author on reasonable request.

## Abbreviations

aPDT: Antimicrobial photodynamic therapy
BHI: Brain heart infusion
Er: YAG: Erbium-doped yttrium aluminum garnet
FEL: Free-electron laser
FT-IR: Fourier transform infrared
IR: Infrared
MIR-FEL: Mid-infrared free electron laser
Nd: YAG: Neodymium-doped yttrium aluminum garnet
TUS: Tokyo University of Science
